# Attention-deficit/hyperactivity disorder, delay discounting, and risky financial behaviors: A preliminary analysis of self-report data

**DOI:** 10.1371/journal.pone.0176933

**Published:** 2017-05-08

**Authors:** Theodore P. Beauchaine, Itzhak Ben-David, Aner Sela

**Affiliations:** 1 Department of Psychology, The Ohio State University, Columbus, Ohio, United States of America; 2 Department of Finance, The Ohio State University, Columbus, Ohio, United States of America; 3 Warrington College of Business, Department of Marketing, University of Florida, Gainesville, Florida, United States of America; University Children's Hospital Tuebingen, GERMANY

## Abstract

Delay discounting—often referred to as hyperbolic discounting in the financial literature—is defined by a consistent preference for smaller, immediate rewards over larger, delayed rewards, and by failure of future consequences to curtail current consummatory behaviors. Previous research demonstrates (1) excessive delay discounting among individuals with attention-deficit/hyperactivity disorder (ADHD), (2) common neural substrates of delay discounting and hyperactive-impulsive symptoms of ADHD, and (3) associations between delay discounting and both debt burden and high interest rate borrowing. This study extends prior research by examining associations between ADHD symptoms, delay discounting, and an array of previously unevaluated financial outcomes among 544 individuals (mean age 35 years). Controlling for age, income, sex, education, and substance use, ADHD symptoms were associated with delay discounting, late credit card payments, credit card balances, use of pawn services, personal debt, and employment histories (less time spent at more jobs). Consistent with neural models of reward processing and associative learning, more of these relations were attributable to hyperactive-impulsive symptoms than inattentive symptoms. Implications for financial decision-making and directions for future research are discussed.

## Introduction

By definition, attention-deficit/hyperactivity disorder (ADHD) is an impairing psychiatric condition. [1] Those with the hyperactive/impulsive and combined presentations in particular experience strong preferences for immediate over delayed rewards [2,3], difficulty inhibiting intemperate behaviors. [4] and prospective vulnerability to increasingly intractable comportment outcomes across development. [5] They also experience academic underachievement and grade retention compared with healthy, age-matched peers. [6] Such outcomes evoke social rejection and stigmatization, resulting in considerable intrapersonal distress. [7,8]

It was once thought that ADHD was a disorder of childhood and adolescence, but research now indicates continued impairment into adulthood. In fact, adults who were diagnosed with ADHD as children report high rates of school drop-out, underemployment, compromised job performance, difficulty maintaining employment, and problems sustaining friendships. [9] Even more troubling, such individuals are prone to impulsive and risky behaviors including substance abuse, self-inflicted injury, and suicide attempts. [10,11] These sequelae are observed even among those who received effective treatment in childhood. [12].

Although understudied to date, impulsive and risky decision making among adults with ADHD also manifest in adverse financial outcomes. Adults with ADHD incur more auto loans, have more difficulty paying bills, and open fewer savings accounts than their peers. [9] In addition, symptoms of ADHD among college students are associated with compulsive credit card use. [10]. Such outcomes, combined with spotty employment histories, likely contribute to high rates of financial distress reported by adults with ADHD. [13]

Neural processing of monetary incentives—including valuations of financial outcomes and decisions about short-term vs. long-term gains and losses—is subserved by functional interactions between the midbrain (mesolimbic) dopamine (DA) system and both the prefrontal and orbitofrontal cortices. Among healthy controls, choices to receive long-term over immediate rewards are associated with frontal activation, whereas choices to receive immediate over long-term rewards are associated with mesolimbic activation. [14] In addition, when previously favorable reward contingencies become unfavorable, neural activity migrates from mesolimbic structures to cortical structures, which inhibit consummatory impulses. [15]. This top-down regulation allows individuals to revalue changing reward contingencies, then choose among more favorable, long-term options. [16]

Abnormalities in mesolimbic responding to incentives, and deficiencies in top-down neural control over subcortically-mediated impulses, are hallmarks of the hyperactive-impulsive and combined presentations of ADHD. [5] Those with the hyperactive-impulsive and combined presentations ADHD exhibit deficient mesolimbic reactivity to incentives [17], persist in reward-seeking behavior after reward contingencies turn unfavorable, and fail to migrate neural activity from midbrain structures to cortical structures when short-term rewards are no longer available. [15] These abnormalities in incentive processing give rise to both associative learning deficits and delay discounting. [18–21] Delay discounting refers to the tendency to devalue magnitudes of delayed rewards and/or over-value magnitudes of immediate rewards, which results in less advantageous decision-making. In turn, associative learning deficits and delay discounting predict adverse financial outcomes, including smaller debt-to-asset ratios. [22]

Outside the laboratory, delay discounting manifests in failure of temporally deferred consequences to curtail present behavior, since long-term consequences are also discounted. Accordingly, delay discounting is often referred to as ‘present bias.’ Those who exhibit present bias tend toward impulsive consummatory behaviors, with minimal or no consideration of future consequences—core features of the hyperactive-impulsive and combined presentations of ADHD. [23]. Perhaps unsurprisingly, delay discounting is also associated with addictive behaviors, including alcohol use, tobacco use, drug use, and gambling. [24,25] All of these conditions are characterized by mesocorticolimbic dysfunction, as described above.

This discussion suggests that financial outcomes of those with ADHD may be affected more adversely than limited data collected to date indicate. We hypothesize that delay discounting—referred to as hyperbolic discounting in the economics literature—will be reflected in associations between ADHD symptoms and previously unevaluated aspects of financial wellbeing. In theory, impulsive consummatory behaviors characteristic of delay discounting should lead to low savings and high borrowing [26,27], including excess credit card use, pawn shop use, and payday lending. [28]. Pawn shops provide short-term loans that are typically repaid within 30 days and require collateral. A lendee might bring in jewelry, which the pawn shop keeps in exchange for cash of significantly less value. The lendee either pays the loan back at a high interest rate (up to 240% APR in some U.S. states) or the pawn shop sells the collateral for profit. In contrast, payday loans are a high interest rate form of short-term lending that usually do not require collateral. Loans are typically due within 30 days, when the lendee can chose to repay in full, or carry the loan forward with an interest-only payment. Annual percentage rates are up to 700% in some U.S. states. Interestingly, when payday borrowers are forced to think about rather than discount future interest rates, their use of payday loans declines. [29] This suggests that payday borrowers ignore future consequences and focus on current transactions, consistent with delay discounting/hyperbolic discounting interpretations.

We evaluate correspondences between ADHD symptoms, measures of delay discounting, and several financial outcomes, including credit card use (carrying balances, late payments, interest rates), amount of savings, and use of extremely high interest rate borrowing (payday loans, pawn shops). Well replicated findings of preference for immediate over long-term rewards suggest that hyperactive-impulsive symptoms of ADHD should predict present bias, more credit card use with less favorable terms, less savings, and more use of extremely high interest lending. As described below, we controlled statistically for a number of possible confounds, including age, education level, income, sex, and substance use. The latter exerts strong effects on financial outcomes, and is associated with both delay discounting and ADHD (see above).

## Method

### Participants

Procedures were reviewed and approved by the University of Florida Institutional Review Board. Informed consent was obtained online, before participants answered survey questions. All 544 participants (mean age 35.3 years, 46% women) were recruited through Amazon’s Mechanical Turk (MTurk), an online labor market comprised of over 100,000 workers in over 100 countries. MTurk is commonly used by social scientists, who post surveys and experiments for workers to choose from. Participation was restricted to respondents located in the U.S. (91.2% native English speakers). Consistent with standard recommendations [30] we also restricted participation to those whose prior session completion rates were 97% or higher. The average completion time was 7.69 min (*SD* = 2.44; range = 2.82–90.48; median = 7.25; excluding the extreme 5% had no effect on the results). Consistent with prevailing MTurk compensation norms, we paid respondents $0.50 for participating. Data quality from MTurk compare favorably with American college samples. [31] Further details are reported elsewhere. [30,32]

### Online questionnaire

#### Demographics

The following demographic information was collected: (1) age (years); (2) sex; (3) English as native language (yes, no); (4) level of education (middle school through doctoral); (5) annual income (<$10,000 to > $120,000); (6) currently employed (yes, no); (7) longest time with a single employer (years); and (8) number of different employers in the past five years.

#### ADHD symptoms

Respondents were asked to endorse or reject, as self-descriptors, each of the 18 symptoms of ADHD in the *Diagnostic and Statistical Manual of Mental Disorders—fifth edition*. [1] These include 9 symptoms of hyperactivity/impulsivity and 9 symptoms of inattention. Symptoms were summed to form hyperactivity/impulsivity, inattention, and combined scores. This approach yields internal consistencies, as assessed by coefficient α, in the .9 range. [33]

#### Delay discounting/Present bias

Present bias was evaluated by asking participants which option they preferred given the following choices: (1) $120 in 1 week vs. $120 in 1 year; (2) $120 in 1 week vs. $137 in 1 year; (3) $120 in 1 week vs. $154 in 1 year; (4) $120 in 1 week vs. $171 in 1 year; (5) $120 in 1 week vs. $189 in 1 year; (6) $120 in 1 week vs. $206 in 1 year; (7) $120 in 1 week vs. $223 in 1 year; and (8) $120 in 1 week vs. $240 in 1 year. Present bias scores (1–8) were assigned based on the highest among these options in which respondents chose the smaller present reward rather than the larger future reward. Similar measures are common in the literature. [34]

#### Self-control

Convergent validity of associations between impulsivity and financial outcomes was evaluated using the Self Control Scale (SCS). [35] Internal consistencies of the SCS also approach .9. High SCS scores are associated with positive psychological adjustment in a number of domains, whereas low scores are associated with impulse control problems, debt burden, and compulsive buying behaviors. [36] Including the SCS enabled us to evaluate financial outcomes across a broader range of individual differences in self-control than symptoms of ADHD capture.

#### Substance use

Substance use was evaluated using the National Institute on Drug Abuse Quick Screen. [37] The measure yields excellent sensitivity (100%) and good specificity (73.5%) in clinical settings. Respondents were asked about frequency of alcohol, tobacco, non-medical prescription drug, and illegal drug use. Ratings were rendered 5 point scales (1 = never to 5 = daily), which were summed across substances to derive a total score.

#### Financial outcomes

Financial outcomes were assessed with a series of questions including (1) do you have a credit card (yes, no); (2) if so, how often are you late on credit card payments (never, yearly, every couple of months, monthly); (3) do you carry a credit card balance (yes, no); (4) if so, at what interest rate (don’t know, 5–10%, 10–15%, >15%); (5) what fraction of your income do you save (0%, <10%, 10–30%, over 30%); (6) have you used payday loans in the past five years (never, once or twice, several times, almost monthly); (7) if so, what is the average loan amount (<$300, $300-$500, $500-$1000, >$1000); (8) have you used pawn shop services in the past five years (never, once or twice, several times, almost monthly); (9) if so, what is the total value of items you’ve pawned in the past five years (<$1000, $1000-$5000, $5000-$10,000; >$10,000); and (10) what is your approximate current debt in dollars?

## Results

Demographic characteristics and variable correlations are presented in Table 1. Although specifics can be gleaned therein, a few findings warrant elaboration. First, ADHD scores were associated negatively with age, years with current employer, and self-control, yet positively with number of jobs held in the past five years, credit card balances carried, credit card late payments, use of pawn services, substance use, and present bias. All of these findings are consistent with previous research and/or a priori expectations, as outlined above. In addition, present bias was associated with credit card late payments, credit card interest rates, payday loan amounts, lower incomes, and less education. Finally, most financial outcomes were correlated with one another, and (inversely) with self-control.

**Table 1:** Descriptive statistics and variable correlations

Variable	1		2			3		4		5		6		7		8		9		10		11		12		13		14		15		16		17		18		19		20	
1. ADHD symptom score		-	-.14		**	.08		-.09		.00		.00		-.15	**	.14	**	-.10		.02		.13	**	.19	**	.15		.07		.09		.14	**	.06		.15	**	-.53	**	.11	*
2. Age			-			.10		.13	*	.06		.01		.68	**	-.17	**	-.06		.09		-.01		-.13	*	.24		-.11	*	-.08		-.13	*	-.13	*	-.06		.31	**	-.15	**
3. Sex						-		-.02		.01		-.15	**	.02		.02		-.24	**	.02		.03		-.02		.21	**	-.09		-.09		-.10		-.13	*	-.09		.04		-.01	
4. Income								-		.26	**	.14	**	.25	**	.03		.19	**	.13	*	-.01		-.12	*	-.01		-.09		-.09		-.11	*	-.10		-.04		.18	**	-.15	**
5. Education										-		.18	**	.04		.10		.12	*	.07		-.05		.01		-.14		-.04		-.01		-.06		-.01		-.17	**	.11	*	-.18	**
6. Currently Employed?												-		.15	**	.31	**	.15	**	.10		.13	*	.09		.01		.10		.12	*	.09		.08		.11	*	-.01		-.06	
7. Years with current employer														-		-.10		.03		.10		.03		-.11		.19	**	-.09		-.10		-.13	*	-.12	*	-.06		.26	**	-.08	
8. Number of jobs (past 5 years)																-		.04		.00		.04		.13	**	-.08		.02		.04		.03		-.03		.05		-.09		.04	
9. Savings (% of income)																		-		-.08		-.17	**	-.15	**	-.26	**	.00		.00		.00		.09		-.09		.16	**	-.07	
10. Debt																				-		.04		-.15	*	.20	**	-.33	**	-.26	**	-.26	**	-.37	**	-.01		-.06		.04	
11. Credit card balance																						-		.31	**	.18	**	.14	*	.16	*	.12	*	.11		.16	**	-.15	*	.19	**
12. Credit card late payments																								-		.09		.40	**	.32	**	.44	**	.35	**	.22	**	-.28	**	.17	**
13. Credit card interest rate																										-		-.03		-.23		-.02		-.05		.04		-.09		-.01	
14. Used payday loans (past 5 years)?																												-		.80	**	.55	**	.55	**	.23	**	-.25	**	.10	
15. Payday loan amount (past 5 years)																														-		.50	**	.51	**	.12	*	-.24	**	.12	*
16. Used pawn services (past 5 years)?																																-		.80	**	.26	**	-.24	**	.10	
17. Amount pawned (past 5 years)																																		-		.20	**	-.20	**	.07	
18. Substance use																																				-		-.27	**	.08	
19. Self-control																																						-		-.14	**
20. Present bias																																								-	
*N*	544		544			543		543		544		532		544		543		524		544		409		413		248		536		536		542		542		544		544		544	
mean *SD*	3.52 2.98		35.27 11.48			- -		- -		- -		- -		6.84 5.93		2.34 1.13		1.41 0.82		$18.9k $7.4k		- -		- -		- -		- -		- -		- -		- -		6.41 2.72		3.55 0.62		5.86 2.44	

*Notes*. Sex was coded male = 1, female = 2. Means and standard deviations are reported for interval and ratio scales only. 46% of participants were women; average income was between $40,000 and $50,000; average education level was between 2 and 4 years of college; 82.9% of participants were currently employed; 47.2% of participants reported carrying a credit card balance; 19.6% of participants reported at least one late credit card payment; average credit card interest rate was between 10% and 30%; 14.0% of participants used payday loans in the past five years (among whom, the average amount was near $500); 16.9% of participants used pawn services in the past five years (among whom, the average total amount pawned was near $1000).

*p≤.01.

**p≤.001.

We executed a series of multiple linear regressions (MLRs) to assess relations between ADHD scores and employment histories, financial outcomes, use of extremely-high interest rate borrowing, self-control, and present bias, controlling for age, income, sex, education, and substance use. Language was not covaried given that the vast majority of participants were native English speakers. In MLR, coefficients for each variable reflect their significance when entered into the regression equation last. They therefore assess independent effects of each variable, over-and-above all others in the equation. Thus, there was no need for stepwise entry, which is appropriate for variable sets. [38].

In the first set of MLRs, ADHD total scores were evaluated. Results are presented in Table 2. ADHD symptoms were associated with late credit card payments, carrying credit card balances, number of jobs held, use of pawn services, debt, poor self-control, and present bias, over-and-above effects of age, income, sex, education, and substance use. Of note, even though substance use was associated with almost all of the financial variables assessed, ADHD scores provided independent prediction of most of these outcomes.

**Table 2:** Multiple regressions evaluating relations between ADHD scores and employment histories, financial outcomes, use of extremely-high interest rate borrowing, self-control, and present bias

	Criterion variable																											
Predictor	late cc payments		cc balance		cc interest rate		currently employed		number of jobs		length with employer		savings % of income		use pay- day loans		payday amount		use pawn services		amount pawned		debt (log)		self- control		present bias	
ADHD symptom score	.137	**	.113	*	.173	**	-.004		.112	***	-.040		-.063		.028		.066		.093	*	.015		.130	**	-.472	***	.081	*
Age	-.091		.015		.255	***	-.008		-.162	***	.652	***	-.075		-.082		-.050		-.084	*	-.094	*	.071		.210	***	-.119	**
Income	-.111	*	.013		.020		.101	*	.031		.171	***	.169	***	-.070		-.087	*	-.083		-.097	*	.101		.100	**	-.091	*
Sex	-.010		.032		.178	**	-.139	***	.030		-.034		-.222	***	-.062		-.078		-.078		-.103	*	.109	*	.039		.004	
Education	.082		-.030		-.155	**	.180	***	.112	**	-.046		.063		.021		.032		.004		.050		.082		.038		-.141	**
Substance use	.201	***	.141	***	.022		.133	*	.049		-.018		-.091		.216	***	.103		.232	***	.189	***	.085	*	-.174	***	.031	
*N*	413		408		248		532		542		542		523		535		535		541		541		543		543		542	
model *R*-square	.097	***	.039	**	.148	***	.083	***	.059	***	.487	***	.109	***	.071	***	.037	**	.101	***	.073	***	.061	***	.388	***	.068	***

*Notes*. All coefficients are standardized.

*p≤.05.

**p≤.01.

***p≤.001.

Next, we re-ran the MLRs with hyperactive/impulsive and inattentive symptoms entered separately. Results are presented in Table 3. As outlined above, this analysis was important given strong evidence that (1) the inattentive presentation is distinct etiologically from hyperactive-impulsive and combined presentations [39–43], and (2) only the hyperactive/impulsive presentation is characterized by frontostriatal neural dysfunction, which underlies delay discounting and risky financial decision making (see extended discussion above). As expected, 5 of 8 significant effects linking ADHD to outcome variables were attributable independently to hyperactive/impulsive symptoms, whereas only 1 of 8 was attributable independently to inattentive symptoms. In fact, the only variable that was associated independently with inattention was *self-control*, which accounted for variance in both hyperactive/impulsive symptoms (*b* = -.406), and inattentive symptoms (*b* = -.131).

**Table 3:** Multiple regressions evaluating relations between hyperactivity/impulsivity scores, inattention scores, and employment histories, financial outcomes, use of extremely-high interest rate borrowing, self-control, and present bias

	Criterion variable																											
Predictor	late cc payments		cc balance		cc interest rate		currently employed		number of jobs		length with employer		savings % of income		use pay- day loans		payday amount		use pawn services		amount pawned		debt (log)		self- control		present bias	
ADHD symptom score																												
hyperactive-impulsive	.112	*	.177	**	.109		.075		.099	*	-.066		.003		.073		.078		.050		.001		.154	**	-.131	***	.035	
inattentive	.047		-.042		.076		-.080		.030		.017		-.079		-.039		-.001		.056		.014		.004		-.406	***	.059	
Age	-.077		.030		.231	***	-.008		-.166	***	.649	***	-.079		-.079		-.049		-.084	*	-.095	*	.072		.215	***	-.119	**
Income	-.116	*	.011		.052		.103	*	.034		.171	***	.170	***	-.071		-.086	*	-.083	*	-.097	*	.105	*	.104	**	-.091	*
Sex	-.015		.029		.178	**	-.146	***	.026		-.033		-.229	***	-.062		-.078		-.078		-.103	*	.104	*	.034		.004	
Education	.071		-.038		-.147	**	.169	***	.106	*	-.045		.060		.017		.029		.004		.050		.073		.029		-.140	***
Substance use	.191	***	.133	**	.045		.123	**	.043		-.017		-.095	*	.213	***	.101	*	.232	***	.189	***	.076		-.182	***	.031	
*N*	413		408		248		532		542		542		523		535		535		541		541		543		543		542	
model *R*-square	.096	***	.037	**	.140	***	.088	***	.060	***	.488	***	.114	***	.075	***	.039	**	.101	***	.074	***	.066	***	.402	***	.068	***

*Notes*. All coefficients are standardized.

*p≤.05.

**p≤.01.

***p≤.001.

## Discussion

We evaluated correspondences between self-reported ADHD symptoms, delay/hyperbolic discounting, and financial outcomes among a large sample of adults, by assessing both hyperactive-impulsive and inattentive symptoms, present bias, savings, debt, use of very high interest rate borrowing, and late payments—controlling for a host of extraneous influences. Debt burden, late credit card payments, and high interest rate borrowing (e.g., payday loans, pawnshop use) were associated with one another. Many of these outcomes were also associated with present bias and hyperactive-impulsive symptoms of ADHD, although effect sizes were generally modest. Nevertheless, findings are likely meaningful given that (1) they were significant over-and-above effects of substance use, income, and education—all of which are associated strongly with both ADHD and delay discounting; and (2) they were more specific to hyperactive-impulsive symptoms, consistent with hypotheses derived from neural accounts of both ADHD and delay discounting.

In contrast to specific associations between financial outcomes and hyperactive-impulsive symptoms, present bias was associated with total ADHD scores, but not independently with either inattentive or hyperactive-impulsive symptoms. This remained the case when covariates were removed from the model. With only hyperactive-impulsive and inattentive symptoms entered as predictors of present bias, the regression equation was significant, *F*(2,543) = 3.27, *p* < .04, even though neither hyperactivity-impulsivity nor inattention provided independent prediction, both *b*s≤.08, both *p*s≥.12. Thus, although relations between financial outcomes and ADHD were specific to hyperactive-impulsive symptoms, relations between present bias and ADHD were not.

In contrast, the strong relation between ADHD symptoms and self-control scores (*r* = -.53, *p* < .001) derived from hyperactive-impulsive and inattentive symptoms, both *b*s≤-.13, both *p*s≤.001. Collectively, differential associations among variables indicate that our measures of ADHD symptoms, present bias, and self-control were not redundant.

The effect size we observed for the relation between present bias and ADHD symptoms was small, *η* = .11. This effect size is about half that reported elsewhere in a recent meta-analysis of 21 studies (*N* = 3913) of delay-discounting in ADHD. [44] There are two likely explanations for this discrepancy. First, all of the studies in the meta-analysis were case-control designs. Thus, those with formal diagnoses of ADHD were compared with controls. Second, although delay/hyperbolic discounting is assessed in many studies the same way it was measured here, computerized tasks are also common, which may yield larger effect sizes because participants usually respond across greater numbers of trials. For example, in one recent study, participants were presented with 91 discounted choices [21], as opposed to 8 in our study. Their effect size was three times what we observed.

The biggest limitations of this study stem from convenience sampling using very brief measures of ADHD, present bias, and financial outcomes. All data we collected were self-report, and therefore may suffer from systematic response biases and halo effects. Anonymity softens such effects, and many participants reported significant substance use, which suggests a reasonable level of candor. Nevertheless, future research should include more detailed assessments of ADHD symptoms, more extensive indices of delay/hyperbolic discounting, and more objective measures of financial outcomes. Indeed, only 248 of 409 participants who carried credit card balances were able to report their interest rates. Given issues with inattention among those with ADHD, such lack of awareness of financial details is more likely, which may have resulted in under-reporting and smaller effect sizes.

These limitations notwithstanding, our findings suggest that relations between hyperactivity-impulsivity and important financial outcomes generalize to broader samples. We view our results as an important step toward improving our understanding of sources of delay discounting and their implications for financial wellbeing. Our findings connect hyperactive-impulsive ADHD symptoms—which mark pursuit of short-term rewards and associated delay discounting—to real financial decisions and outcomes. Furthermore, our results demonstrate that hyperactivity-impulsivity—not inattention—is the main driver of these effects.
